# Dietary *n-3* and *n-6* polyunsaturated fatty acids, the *FADS* gene, and the risk of gastric cancer in a Korean population

**DOI:** 10.1038/s41598-018-21960-3

**Published:** 2018-02-28

**Authors:** Sunghee Lee, Jeonghee Lee, Il Ju Choi, Young-Woo Kim, Keun Won Ryu, Young-Il Kim, Jeongseon Kim

**Affiliations:** 1 Department of Cancer Biomedical Science, Graduate School of Cancer Science and Policy, National Cancer Center, Goyang-si, Gyeonggi-do South Korea; 2 Center for Gastric Cancer, National Cancer Center, Goyang-si, South Korea; 3 Department of Food and Nutrition, College of Health Science, Kangwon National University, Samcheok-si, Gangwon-do South Korea

## Abstract

*n-3* polyunsaturated fatty acids (PUFAs) and *n-6* PUFAs are reported to have immunomodulatory effects, but few studies have examined these functions. Thus, we examined whether dietary *n-3* and *n-6* PUFAs are associated with the risk of gastric cancer and further investigated whether fatty acid desaturases 1 and 2 (*FADS1* and *FADS*2) modify this association. In a case-control study, 1,464 participants (402 cases and 1,062 controls) were enrolled. A semi-quantitative food frequency questionnaire was utilized to measure dietary PUFA intake. Genotyping was performed using the Axiom® Exome 319 Array. Multivariable logistic models were established after adjusting for confounding variables. The risk of gastric cancer was significantly decreased among participants who had the highest tertile intake of docosahexaenoic acid (DHA), an *n*-*3* PUFA, even after adjusting for covariates [odds ratios (OR) = 0.72, 95% confidence intervals (95% CIs) = 0.53–0.99]. However, no significant interaction according to *FADS1* rs174546 or *FADS2* rs174583 was observed. In conclusion, we observed a significant inverse association between dietary DHA and the risk of gastric cancer but found that *FADS1* rs174546 and *FADS2* rs174583 did not modify the association between dietary *n-3* or *n-6* PUFAs and gastric cancer risk.

## Introduction

Gastric cancer is the fifth most prevalent cancer and the third leading cause of cancer mortality worldwide^1^. Gastric cancer is the most common cancer among Korean men^2^. The high prevalence of *Helicobacter pylori (H. pylori)* infection of approximately 54.4% in Korean adults substantially contributes to gastric cancer^3^. The relatively high infection rate of *H. pylori* leads to chronic mucosal infection and inflammation^4^, thereby increasing the individual’s vulnerability for gastric carcinogenesis. Data on various dietary approaches, including ones involving *n-3* polyunsaturated fatty acids (PUFAs), to reduce chronic inflammation related to increased risk of gastric cancer have been increasing^5^.

*n-3* PUFAs and *n-6* PUFAs exhibit immunomodulatory effects. Specifically, the eicosanoids converted from 20-carbon PUFAs act to modulate inflammatory and immune responses to affect cell growth and differentiation^6^. Specifically, *n-3* PUFAs play important anti-inflammatory roles, whereas *n-6* PUFAs, particularly arachidonic acid (AA), exert pro-inflammatory effects^7^. In addition, *n-3* PUFAs reduce the risk of cancer through the inhibition of AA, which produces pro-inflammatory eicosanoids and promotes carcinogenesis and disease progression^7^. Furthermore, the eicosanoids of *n-3* PUFAs exhibit an inhibitory effect that suppresses those of AA, resulting in inhibition of carcinogenesis via the modulation of inflammation^7^. Given these reciprocal actions, the ratio of dietary *n-6* to *n-3* PUFAs has been examined. Higher *n-6* to *n-3* PUFAs ratios are associated with an increased risk of carcinogenesis^8,9^.

Several genome-wide association studies (GWASs) have examined genetic loci significantly associated with *n-3* and *n-6* PUFAs and identified several single nucleotide polymorphisms (SNPs), including fatty acid desaturases 1 and 2 (*FADS1* and *2*)^10^. These GWASs have been replicated in Singaporean^11^, Chinese and European- populations^12^ and in multi-ethnic meta-analyses^13,14^.

Previous epidemiological studies have demonstrated that *n-3* PUFAs, especially marine-derived *n-3* fatty acids^15^, reduce the risk of cancer^6,16^, whereas dietary *n-6* PUFAs have no association with increased cancer risk^17,18^. However, the association between the risk of gastric cancer and dietary *n-3* PUFAs has never been studied. In addition, genetic variants associated with fatty acid metabolism might modify the beneficial anti-tumorigenic effects of PUFAs.

Therefore, we examined whether dietary *n-3* and *n-6* PUFAs are associated with the risk of gastric cancer and sought to determine whether *FADS1* and *FADS2* modify the association between PUFAs and the risk of gastric cancer. For example, dietary *n-3* fatty acids such as α-linolenic acid (ALA, c18:3[*n-3*]), stearidonic acid (c18:4[*n-3*]), eicosatetraenoic acid (c20:4[*n-3*]), eicosapentaenoic acid (EPA, c20:5[*n-3*]), docosapentaenoic acid (DPA, c22:5[*n-3*]), docosahexaenoic acid (DHA, c22:6[*n-3*]) were examined. In addition, dietary *n-6* fatty acids including linoleic acid (LA, c18:2[*n-6*]), eicosadienoic acid [c20:2(*n-6*)], dihomo-γ-linolenic acid (c20:3[*n-6*]), AA (c20:4[*n-6*]) were measured. We hypothesized that dietary *n-3* PUFAs were inversely associated with the risk of gastric cancer, whereas dietary *n-6* PUFAs would increase this risk. In addition, we hypothesized that the risk of gastric cancer associated with dietary *n-3* and *n-6* PUFAs would vary according to *FADS1* or *FADS2* genetic variants.

## Results

### General participant characteristics

Table 1 shows the general characteristics of the study participants with and without gastric cancer. Gastric cancer cases exhibited an increased proportion of men compared with the control group (*p* < 0.001). However, body mass index did not differ between the groups. Smoking, measured as pack-years, was higher among the cases. Alcohol consumption, based on ethanol amount in grams per day, was significantly greater among cases than controls. The prevalence of *H. pylori* infection was higher among cases (91.79%) than controls (60.64%). The cases were less likely to exercise regularly (35.57%) than controls (53.67%). The cases were significantly more likely to have a family history of gastric cancer than controls (21.89% vs. 13.37%). With respect to dietary intake, the cases consumed more total calories than controls (*p* < 0.001). The cases consumed more ALA than the controls (*p* = 0.002). No difference was observed between the cases and controls regarding the intake of EPA, DHA and the sum of both. However, the cases consumed significantly more dietary *n-6* PUFAs, including LA and AA, than the controls (*p* < 0.001 and *p* < 0.001, respectively).

**Table 1:** General characteristics of the study participants (n = 1,464).

	Case (n = 402)	Control (n = 1,062)	*p*-value
Age, years	55.27 ± 10.91	52.03 ± 8.60	<0.001
Women	138 (34.33)	557 (52.45)	<0.001
Body mass index, kg/m^2^	23.75 ± 3.10	23.79 ± 2.93	0.844
Smoking, pack-years^*^	16.74 ± 19.66	9.93 ± 77.48	<0.001
Drinking, ethanol amount, g/day^*^	19.46 ± 36.85	9.33 ± 19.96	<0.001
*Helicobacter pylori*, positive	369 (91.79)	644 (60.64)	<0.001
Regular exercise	143 (35.57)	570 (53.67)	<0.001
Family history of gastric cancer	88 (21.89)	142 (13.37)	<0.001
**Dietary intake**			
Total energy intake, kcal/day	1920.44 ± 650.95	1716.48 ± 577.86	<0.001
Total fat intake, g/day	35.19 ± 22.52	31.91 ± 18.20	0.009
***n-3*** **polyunsaturated fatty acids**			
α-Linolenic acid (ALA), mg/day	355.13 ± 219.74	316.19 ± 182.20	0.002
Eicosapentaenoic acid (EPA), mg/day^*^	86.37 ± 96.00	82.90 ± 101.68	0.259
Docosahexaenoic acid (DHA), mg/day^*^	172.05 ± 208.67	168.48 ± 224.29	0.437
Sum of both EPA + DHA, mg/day^*^	258.42 ± 303.83	251.37 ± 325.66	0.366
***n-6*** **polyunsaturated fatty acids**			
Linoleic acid (LA), mg/day	4579.01 ± 2442.08	4067.31 ± 2122.02	<0.001
Arachidonic acid (AA), mg/day	16.59 ± 14.52	13.05 ± 11.80	<0.001

Mean ± S.D. or n (%); *p-value from the Wilcoxon test.

### Association between PUFAs and risk of gastric cancer

Table 2 shows the association between the tertile ranges of dietary *n-3* and *n-6* PUFA intakes and the risk of gastric cancer. The highest tertile of DHA intake showed a significantly reduced risk of gastric cancer compared with the lowest tertile (OR = 0.72, 95% CIs = 0.53–0.99, *p*-value for trend = 0.039), even after adjusting for age, gender, total caloric intake, body mass index, smoking (pack-years), drinking (ethanol amount), physical activity, *H. pylori* infection and family history of gastric cancer. The sum of marine-derived fatty acids, EPA + DHA, showed a borderline significant trend for the reduced risk of gastric cancer as the intake increased (*p*-value for trend = 0.045). In contrast, the highest tertile of AA intake showed an increased but not significant risk of gastric cancer compared with the lowest tertile. We performed separate analyses for men and women and did not perform a gender-based adjustment of the model presented in Supplementary Table 1. Males who consumed the highest levels of DHA exhibited a significantly decreased risk of gastric cancer (OR = 0.64, 95% Cis = 0.42–0.99, *p*-value for trend = 0.042).

**Table 2:** Association between dietary *n-3* and *n-6* fatty acid intake with the risk of gastric cancer (n = 1,464).

	Tertile range of dietary fatty acids (mean ± S.D.)			*p*_trend_
	T1	T2	T3	
	Odds Ratios (95% Confidence Intervals)			
***n-3*** **Polyunsaturated fatty acids, mg/day**				
α-Linolenic acid (ALA)	200.74 ± 88.71	318.14 ± 129.82	470.91 ± 231.64	0.403
	1.00 (Ref)	0.87 (0.64, 1.19)	0.88 (0.64, 1.20)	
Eicosapentaenoic acid (EPA)	22.75 ± 14.05	60.50 ± 28.30	171.19 ± 132.56	0.112
	1.00 (Ref)	0.88 (0.65, 1.21)	0.78 (0.57, 1.06)	
Docosahexaenoic acid (DHA)	38.41 ± 29.10	119.03 ± 65.64	359.48 ± 294.71	0.039
	1.00 (Ref)	0.76 (0.56, 1.04)	0.72 (0.53, 0.99)	
Sum of both EPA + DHA	61.52 ± 41.33	177.43 ± 93.09	530.81 ± 425.13	0.045
	1.00 (Ref)	0.76 (0.56, 1.05)	0.73 (0.53, 0.99)	
***n-6*** **Polyunsaturated fatty acids, mg/day**				
Linoleic acid (LA)	2785.99 ± 1178.20	4092.09 ± 1496.51	5860.19 ± 2624.21	
	1.00 (Ref)	1.14 (0.84, 1.55)	0.80 (0.58, 1.11)	0.203
Arachidonic acid (AA)	5.52 ± 3.66	11.57 ± 5.44	24.11 ± 15.71	
	1.00 (Ref)	1.14 (0.82, 1.59)	1.33 (0.96, 1.83)	0.083

Tertile ranges of energy-adjusted dietary fatty acids; mean ± S.D. values are indicated as the values before energy adjustment. Adjusted for age, gender, total caloric intake, body mass index, smoking (pack-years), drinking (ethanol amount), physical activity, *H. pylori* infection and family history of gastric cancer.

### Association between genetic polymorphisms and gastric cancer risk

Table 3 presents the association between the two SNPs on genes *FADS1* rs174546 and *FADS2* rs174583 and the risk of gastric cancer. Individuals with gastric cancer showed no significant association with *FADS1* and *FADS2* genes.

**Table 3:** Association between genetic polymorphisms and gastric cancer risk (n = 1,464).

		Case	Control	*p*-value	OR (95% CI)
		n (%) 402 (27.46)	n (%) 1,062 (72.54)		
***FADS1*** rs174546	CC	192 (47.76)	475 (44.73)	0.298	1.12 (0.89, 1.42)
	TC/TT	210 (52.24)	587 (55.27)		1.00 (Ref)
***FADS2*** rs174583	CC	189 (47.01)	462 (43.50)	0.228	1.15 (0.91, 1.46)
	TC/TT	213 (52.99)	600 (56.50)		1.00 (Ref)

ORs (odds ratios) and 95% CIs (95% confidence intervals) were adjusted for age and gender.

*FADS1* rs174546 and *FADS2* rs174583 are located on chromosome 11. The minor allele frequencies (MAFs) were approximately 0.03 and 0.34. The characteristics are listed in detail in Supplementary Table 2.

### Modifying effects of FADS genes on the associations between the risk of gastric cancer and EPA + DHA and AA

The modifying effects of the genes *FADS1* and *FADS* 2 are presented in Table 4. A separate analysis was performed for men and women, and this analysis did not involve gender adjustment of the model presented in Supplementary Table 3. No significant modifying effect was observed.

**Table 4:** Modifying effects of *FADS* genetic variants on the risk of gastric cancer associated with EPA, DHA, and AA (n = 1,464).

		Case/Control	**EPA**			*p*_interaction_	**DHA**			*p*_interaction_	**AA**			*p*_interaction_
			OR (95% CI)				OR (95% CI)				OR (95% CI)			
			T1	T2	T3		T1	T2	T3		T1	T2	T3	
***FADS1*** **rs174546**	CC	192/475	1.17 (0.76, 1.80)	0.93 (0.59, 1.45)	0.88 (0.56, 1.39)	0.859	1.06 (0.69, 1.62)	0.81 (0.52, 1.27)	0.78 (0.50, 1.22)	0.699	0.93 (0.58, 1.49)	0.99 (0.62, 1.57)	1.64 (1.05, 2.56)	0.102
	TC/TT	210/587	1.00 (Ref)	0.97(0.63, 1.49)	0.80(0.52, 1.23)		1.00 (Ref)	0.76 (0.49, 1.16)	0.71 (0.46, 1.09)		1.00 (Ref)	1.23 (0.78, 1.92)	1.05 (0.68, 1.62)	
***FADS2*** **rs174583**	CC	189/462	1.20 (0.78, 1.85)	0.96 (0.61, 1.50)	0.94 (0.60, 1.47)	0.988	1.09 (0.71, 1.67)	0.84 (0.54, 1.31)	0.83 (0.53, 1.30)	0.561	0.97 (0.60, 1.55)	1.05 (0.66, 1.66)	1.71 (1.10, 2.67)	0.108
	TC/TT	213/600	1.00 (Ref)	0.96 (0.63, 1.47)	0.77 (0.50, 1.19)		1.00 (Ref)	0.76 (0.49, 1.15)	0.69 (0.45, 1.05)		1.00 (Ref)	1.21 (0.77, 1.88)	1.06 (0.69, 1.62)	

ORs (odds ratios) and 95% CIs (95% Confidence Intervals) were adjusted for age, gender, total caloric intake, body mass index, smoking (pack-years), drinking (ethanol amount), physical activity, *H. pylori* infection and family history of gastric cancer. *p*_interaction_ is the *p-*value for the interaction. EPA, eicosapentaenoic acid; DHA, docosahexaenoic acid; AA, arachidonic fatty acid.

## Discussion

In a case-control study of 1,464 participants (402 cases and 1,062 controls), we observed a significant inverse association between DHA and the risk of gastric cancer. However, we did not identify a significant modifying effect according to *FADS1* rs174546 or *FADS2* rs174583 genetic variants on the association between *n-3* or *n-6* PUFAs and the risk of gastric cancer.

The significant inverse associations among the intake of a marine-derived *n-3* fatty acid, DHA, and the risk of gastric cancer observed in the current study are consistent with the results of previous epidemiological studies. A recent study examining the association between the erythrocyte composition of fatty acids and gastric cancer risk revealed a significant inverse association with *n-3* PUFAs on gastric cancer risk (OR = 0.39, 95% CIs = 0.23–0.68) and with DHA on gastric cancer risk (OR = 0.47, 95% CIs = 0.28–0.79)^19^. Other studies have also demonstrated that *n-3* PUFAs, especially marine-derived *n-3* fatty acids^15^, reduce the risk of cancer^6^. Regarding the association with *n-6* PUFAs, two recent studies demonstrated significant associations between AA intake and *FADS1* among Korean^20^ and Japanese adults^21^. Specifically, in a case-control study of patients with coronary artery disease (CAD) and controls, individuals with the *FADS1* rs174537 T allele showed a reduced risk of CAD with reduced AA^20^. Another study suggested that adults with the *FADS1* rs174547 C allele may have a low conversion rate from LA to AA, based on the high LA and low AA results in the plasma as well as erythrocytes^21^.

The immune-modulatory effect of *n-6* PUFAs, particularly AA, has been controversial given the theoretical pro-inflammatory effect of several eicosanoids from AA. Experimental studies have demonstrated that AA is pro-inflammatory^22^, whereas epidemiological studies have revealed that AA had no effect on all-cause mortality^23^. Furthermore, a recent systematic review and meta-analysis reported that AA showed a significant inverse association with coronary risk based on results of 10 prospective observational studies with 22,948 participants^24^. Moreover, another study demonstrated an inverse association between *n-6* PUFAs and mortality from all causes or coronary heart disease (CHD)^23^, and a 14-year longitudinal follow-up study among women showed that *n-6* PUFAs reduced the risk of CHD^25^. However, these protective effects of *n-6* PUFAs specifically refer to LA, i.e., circulating plasma phospholipid LA^23^ and dietary LA comprised 81% of total *n-6* PUFAs^25^. Furthermore, the results of previous epidemiological studies are consistent with our findings of no effect of *n-6* PUFAs on gastric cancer risk. A recent meta-analysis showed no significant association between either dietary or serum LA on breast cancer risk^26^. A systematic review of observational studies demonstrated that AA was not associated with cancer risk^18^. Overall, the current evidence demonstrates that there is no increased risk of cancer associated with *n-6* PUFAs.

These inconsistent findings may be attributable in part to differences in the percentage or actual amount of serum, plasma phospholipid, or adipose fatty acids as a surrogate measurement of dietary intake. Regarding the relationship between *n-3* PUFAs and cancer risk, although a large body of evidence has demonstrated a protective effect on cancer risk through modulation of inflammation, recent studies have reported that the risk of prostate cancer decreased with serum^27^ but increased with plasma phospholipids^27^. However, the study participants with plasma phospholipids appeared to have insufficient levels of *n-3* PUFAs to detect a favorable effect^28^.

Furthermore, in the current study, we explored whether genes related to fatty acid metabolism play roles as effect modifiers in the association between *n-3* or *n-6* PUFAs and the risk of gastric cancer. However, the findings of the current study revealed no significant modifying effect of genes related to fatty acids. Future studies of dietary fatty acid intake using various biomarkers, such as serum fatty acids, are recommended.

Several molecular mechanisms through which PUFAs might modify the carcinogenic process have been proposed^6^: (1) suppression of AA-derived eicosanoid synthesis; (2) influences on transcription factor activity, gene expression, and signal transduction pathways; (3) alternation of estrogen metabolism; (4) increased or decreased production of free radicals and reactive oxygen species; and (5) insulin sensitivity and membrane fluidity. However, the current knowledge on the potential mechanisms of the carcinogenic actions of PUFAs is somewhat inconsistent. In particular, epidemiological studies have failed to identify an association between PUFAs and cancer risk.

Among the mechanisms addressed above, the most plausible mechanism related to the hypothesis of this study is that one of the more important functions of PUFAs is related to their enzymatic conversion into eicosanoids. The mechanisms through which n-3 PUFAs might reduce the risk of cancer involves their suppressive effect on the biosynthesis of AA-derived eicosanoids. This effect might be achieved at several levels: (1) incorporation into membrane phospholipids; (2) competition of n-3 PUFAs with n-6 PUFAs for desaturases and elongases; and (3) suppression of cyclooxygenase-2 (COX-2) and competition with n-6 PUFAs for COX to form eicosanoids. Another possible hypothesis that COX-2 might play an important role in gastric carcinogenesis was suggested based on several studies that found that COX-2 inhibitors might inhibit the development of gastric cancer^29^. However, the precise mechanisms leading to increased COX-2 expression are still not fully clarified; therefore, the mechanisms underlying the antitumoral action of COX-2 inhibitors remain undefined.

A previous study reported that single nucleotide polymorphisms (SNPs) in *FADS1* and *FADS2* might influence plasma fatty acid profiles, influencing inflammatory processes and factors such as high-sensitivity C-reactive protein (hs-CRP)^30^. In general, the *FADS* gene and its family members *FADS1* and *FADS2* are located on Chr 11q12-q13.1. These genes play important roles in the *n-3* and *n-6* family cascade as delta-5 desaturase (D5D) and delta-6 desaturase (D6D) enzymes that produce double bonds and regulate the unsaturation of fatty acids^31^. The *FADS* genes exhibit individual differences in synthesizing and regulating PUFAs, which affect the metabolism of essential fatty acids and subsequently influence health outcome. However, the effect sizes of the *FADS* genes on the dietary PUFAs were very small in the current study (i.e., less than 10%). Furthermore, eicosanoids produced from 20-carbon PUFAs affect inflammatory and immune responses.

Notably, the relationship between gastric cancer risk and dietary *n-3* PUFAs was examined in a population with a relatively high prevalence (54.4%)^3^ of *H. pylori* infection in this study. The *n-3* and *n-6* PUFAs are essential fatty acids that are indispensable for the structure and fluidity of cell membranes. Western diets, which have an abundance of *n-6* PUFAs, have high *n-6* to *n-3* PUFA ratios^32^. A previous study demonstrated that a high ratio of dietary *n-6* to *n-3* PUFAs increased the risk of prostate cancer^9^. Thus, a high intake of *n-3* PUFAs is advised to reduce this ratio^9^.

These effects suggested that *n-3* and *n-6* fatty acids act in a reciprocal manner. However, recent critics have suggested that the *n-6* to *n-3* PUFA ratio should not be used because it cannot discriminate absolute amounts in intake^33^. Moreover, an individual who consumes a small amount of *n-6* fatty acids can have the same ratio as someone who consumes a large amount of *n-3* fatty acids^33^.

This study has several strengths. The food frequency questionnaire (FFQ) used in the current study to measure dietary PUFAs has been previously validated^34^. Moreover, a previous study confirmed that the FFQ is a valid measure of PUFAs^35^, showing de-attenuation correlation coefficients between the FFQ and a 3-day dietary record of total *n-3* fatty acids, EPA, DHA, and AA of 0.49, 0.48, 0.55, and 0.32, respectively^36^. However, this study also has limitations that should be taken into account when interpreting our findings. First, this case-control study might have a selection bias because the control participants were recruited as cases in a hospital. Second, the sample size of the current study was not sufficiently large to allow histological or anatomical analyses of different characteristics, such as intestinal/diffuse type or cardia/non-cardia type of gastric carcinoma. Third, we did not assess an inflammatory biomarker, for example, hs-CRP. However, since measurements of inflammatory biomarkers are part of our ongoing project, future data will be forthcoming. Fourth, we did not measure serum PUFA levels which would have made it possible to investigate the underlying mechanisms of the conversion of desaturases to carbon metabolites. However, we measured dietary PUFA levels which provide evidence for establishing dietary guidelines for the public. Furthermore, it is worthwhile to observe the effect of dietary PUFAs on gastric cancer modified by FADS SNPs.

In conclusion, this case-control study observed a significant inverse association between *n-3* PUFAs and gastric cancer risk after adjusting for age, gender, total caloric intake, body mass index, smoking, drinking, physical activity, *H. pylori* infection and family history of gastric cancer. Furthermore, we observed no interaction between the *FADS1* rs174546 and *FADS2* rs174583 genetic variants and the association between dietary PUFA intake and risk of gastric cancer.

## Methods

### Study population

Participants were recruited from the National Cancer Center (NCC) in Korea between March 2011 and December 2014. The details of the study enrollment were reported previously^37^. Briefly, cases diagnosed and histologically confirmed within three months as early gastric cancer were identified at the gastric cancer center of the NCC. The early stage of gastric cancer was defined as carcinomas confined to the mucosa or submucosa^38^. Individuals with advanced gastric cancer, another cancer diagnosed over past five years, severe mental disease, systematic disease, or diabetes mellitus, as well as pregnant or breast-feeding women, were excluded. During the same period, controls without gastric cancer were enrolled from a sample of participants who received a health screening examination as part of a benefit program of the National Health Insurance Service at the Center for Cancer Prevention and Detection of the NCC. Korean participants received insurance coverage from a national health insurance service program. Those with gastric or duodenal ulcer, cancer, diabetes mellitus, or previous *H. pylori* treatment were excluded. At enrollment, 1,710 individuals (500 cases and 1,210 controls) agreed to participate. Of those participants, fifty individuals were excluded as a result of providing incomplete dietary data, and nine participants who reported a total energy intake of <500 kcal or >5000 kcal were further excluded due to the implausibility of the data. Of those 1,651 adults, 119 were excluded because they did not provide blood samples. Additionally, seven participants were excluded after a quality control step of genotyping. Of the remaining 1,525 participants, 61 adults were excluded due to missing information regarding body mass index (n = 8), physical activity (n = 12), smoking (n = 31), or family history of gastric cancer (n = 10). Therefore, 1,464 participants were included in the final analyses. Supplementary Table 4 presents a comparison of the characteristics of the individuals analyzed (n = 1465) with those excluded (n = 245). Those who were included in the study were likely to smoke and drink more than the individuals who were excluded. All participants agreed to voluntarily participate in this study and provided written informed consent. All protocols for the current study were approved by the Institutional Review Board (IRB) of the NCC (Number: NCCNCS-11-438). All methods for the current study were in accordance with the guidelines and regulations of the IRB.

### Dietary assessment

A validated semi-quantitative FFQ composed of 106 food items was used to assess the daily intake of the participants. The participants provided their individual average frequency of eating and typical portion sizes in the year preceding the interview. These values were converted to obtain daily nutrient intake values using a scale with nine frequency categories (never or rarely, once a month, twice or three times a month, once or twice a week, three or four times a week, five or six times a week, once a day, twice a day, and three times a day) and three portion size categories (small, medium, and large). The validity and reproducibility of the FFQ were confirmed previously^34^. Daily nutrient intake was calculated using CAN-PRO 4.0 (Computer Aided Nutritional analysis program, Korean Nutrition Society, Seoul, Korea). Total energy intakes of <500 kcal and >5,000 kcal were excluded because they reflect implausible values. Energy intake was adjusted using a residual method^39^.

The following *n-3* and *n-6* PUFAs were examined to determine their daily intake. Marine *n-3* PUFA intake comprised both EPA and DHA. The *n-3* fatty acids ALA (c18:3[*n-3*]), stearidonic acid (c18:4[*n-3*]), eicosatetraenoic acid (c20:4[*n-3*]), EPA (c20:5[*n-3*]), DPA (c22:5[*n-3*]), and DHA (c22:6[*n-3*]) and the *n-6* fatty acids LA (c18:2[*n-6*]), eicosadienoic acid [c20:2(*n-6*)], dihomo-γ-linolenic acid (c20:3[*n-6*]), and AA (c20:4[*n-6*]) were measured.

### Genotyping data

Genotyping processes and quality control (QC) were previously published in detail^3^. All participants underwent DNA genotyping performed using an Axiom® Exome 319 Array from Affymetrix (Santa Clara, CA, USA), including 318,983 SNPs. To ensure QC, the exclusion criteria were applied with a high missing gene call rate (greater than 5%), Hardy-Weinberg equilibrium (*p* < 1 × 10^−7^) with regard to the controls, and a cluster QC plot. The genotyping call rates were greater than 95%. Previous large GWASs in East Asian populations, such as those in China and Singapore identified the candidate SNPs as having the most significant associations with the *FADS1* and *FADS2* genes^10–14^. Hardy-Weinberg equilibrium was assessed, revealing a *p-value* = 0.5509 for rs174546 and *p-value* = 0.3192 for rs174583. A linkage disequilibrium (LD) block was assessed between the two candidate SNPs from each *FADS1* and *FADS2* genes (r-square = 0.94).

### Covariate assessment

All participants completed self-administered questionnaires that included demographic characteristics and smoking/drinking status. For example, the amount of alcohol consumed (in grams per day) and pack-years (average packs per smoking years) were calculated. A rapid urease test (Pronto Dry; Medical Instruments Corporation, Solothurn, Switzerland) was used to histologically ascertain *H. pylori* infection status.

### Statistical analyses

The general characteristics of the participants were analyzed using t-tests for continuous variables and chi-square tests for categorical variables. A non-parametric method using the Wilcoxon test was utilized for several variables with asymmetry distributions. Descriptive characteristics according to the *FADS1* and *FADS2* genotypes were examined. In addition, multivariable logistic regression models were established to investigate the association between dietary PUFAs and the risk of gastric cancer; these models estimated the odds ratios (ORs) and 95% confidence intervals (95% CIs) after adjusting for age, gender, total caloric intake, body mass index, smoking (pack-years), drinking (ethanol amount), physical activity, *H. pylori* infection and family history of gastric cancer. According to the literature review related to the risk factors of gastric cancer, variables were selected as confounding factors in the adjusted model, and these variables were statistically significant in the crude model. A trend toward the risk of gastric cancer was examined in association with tertile ranges of PUFA intake. The associations between the *FADS1* and *FADS2* genotypes and the risk of gastric cancer were investigated after adjusting for age and gender. Furthermore, the interaction terms were examined to investigate whether the *FADS1* and *FADS2* genes modified the associations between the risk of gastric cancer and dietary *n-3* and *n-6* PUFA intakes and the *n-6* to *n-3* PUFA ratio. A two-tailed p-value of < 0.05 was considered significant. Statistical analyses were performed using PLINK version 1.07^40^ and SAS version 9.3 (SAS Institute Inc., Cary, NC, USA).

## Electronic supplementary material

supplementary table
