# Supplementary material for: Dietary n-3 and n-6 polyunsaturated fatty acids, the FADS gene, and the risk of gastric cancer in a Korean population
Source: Sci Rep. 2018 Feb 28;8:3823. doi: 10.1038/s41598-018-21960-3 (PMC5830640; doi:10.1038/s41598-018-21960-3)
Supplement: Supplementary file 1 — supplementary table [file 41598_2018_21960_MOESM1_ESM.doc]

**Dietary *n-3* and *n-6* polyunsaturated fatty acids, the *FADS* gene, and the risk of gastric cancer in a Korean population**

Sunghee Lee^1,3^, Jeonghee Lee^1^, Il Ju Choi^2^, Young-Woo Kim^2^, Keun Won Ryu^2^, Young-Il Kim^2^, Jeongseon Kim*^1^

^1^Department of Cancer Biomedical Science, Graduate School of Cancer Biomedical Science, National Cancer Center, Goyang-si, Gyeonggi-do, South Korea

^2^Center for Gastric Cancer, National Cancer Center, Goyang-si, South Korea

^3^Department of Food and Nutrition, College of Health Science, Kangwon National University, Samcheok-si, Kwangwon-do, South Korea

***Corresponding Author:** Jeongseon Kim

Department of Cancer Biomedical Science, Graduate School of Cancer Biomedical Science, National Cancer Center, 323 Ilsan-ro, Goyang-si, Gyeonggi-do, 10408, South Korea

Tel: 82-31-920-2570; Fax: 82-31-920-2579; E-mail: jskim@ncc.re.kr

**Supplementary Table 1**. Gender-specific association between dietary *n-3* and *n-6* fatty acid intake with the risk of gastric cancer (n=1,464)

|  | **Men**  (n=769) | | | *p*_trend_ | **Women**  (n=695) | | | *p*_trend_ |
| --- | --- | --- | --- | --- | --- | --- | --- | --- |
|  | Tertile range of dietary fatty acids  (mean±S.D.) | | |  | Tertile range of dietary fatty acids  (mean±S.D.) | | |  |
|  | T1 | T2 | T3 |  | T1 | T2 | T3 |  |
|  | Odds Ratios (95% Confidence Intervals) | | |  | Odds Ratios (95% Confidence Intervals) | | |  |
| ***n-3* polyunsaturated fatty acids, mg/day** | | | |  |  |  |  |  |
| α-linolenic acid (ALA) | 1.00 (Ref) | 0.71 (0.46, 1.08) | 0.68 (0.44, 1.04) | 0.072 | 1.00 (Ref) | 1.12 (0.69, 1.83) | 1.20 (0.72, 2.01) | 0.480 |
| Eicosapentaenoic acid (EPA) | 1.00 (Ref) | 0.71 (0.46, 1.08) | 0.72 (0.47, 1.10) | 0.127 | 1.00 (Ref) | 1.11 (0.68, 1.80) | 0.80 (0.48, 1.32) | 0.395 |
| Docosahexaenoic acid (DHA) | 1.00 (Ref) | 0.60 (0.39, 0.92) | 0.64 (0.42, 0.99) | 0.042 | 1.00 (Ref) | 0.98 (0.60, 1.60) | 0.76 (0.46, 1.24) | 0.278 |
| Sum of both EPA+DHA | 1.00 (Ref) | 0.61 (0.40, 0.94) | 0.66 (0.43, 1.01) | 0.055 | 1.00 (Ref) | 0.95 (0.58, 1.56) | 0.74 (0.45, 1.22) | 0.245 |
| ***n-6* polyunsaturated fatty acids, mg/day** | | | | |  |  |  |  |
| Linoleic acid (LA) | 1.00 (Ref) | 0.95 (0.63, 1.44) | 0.79 (0.51, 1.21) | 0.274 | 1.00 (Ref) | 1.53 (0.95, 2.47) | 0.84 (0.49, 1.43) | 0.663 |
| Arachidonic acid (AA) | 1.00 (Ref) | 0.81 (0.52, 1.28) | 1.21 (0.79, 1.87) | 0.291 | 1.00 (Ref) | 1.48 (0.88, 2.49) | 1.37 (0.82, 2.30) | 0.231 |

Tertile ranges of energy-adjusted dietary fatty acids; mean±S.D. values were indicated as the values before energy adjustment. Adjusted for age, total caloric intake, body mass index, smoking (pack-years), drinking (ethanol amount), physical activity, *H. pylori* infection and family history of gastric cancer.

**Supplementary Table 2. Descriptive characteristics of genetic polymorphisms**

| Gene | SNP | Chr^a)^ | Position | Position | Major/Minor allele | MAF^b)^ |
| --- | --- | --- | --- | --- | --- | --- |
| ***FADS1*** | rs174546 | 11 | ACTATAGTGGCATTGTCCCTCAAGCTCCCCTCTGC[C/T]TTGGCTCCAGAGTCTTCCTCCTCTTCTTCCAGACT | 61569830 | C/T | 0.326 |
| ***FADS2*** | rs174583 | 11 | CGCAGCGAGCAGCTTGCCTGGCCCTGAGCCTGAAG[C/T]GGCCTGAGAACCTGGTCTCTGTCCAGAATCTCAGG | 61609750 | C/T | 0.335 |

1. Chr., Chromosome; b) MAF, minor allele frequency

**Supplementary Table 3.** Gender-specific modifying effects of *FADS* genetic variants on gastric cancer associated with EPA, DHA, and AA (n=1,464)

|  | | | **Men** | | | *p*_interaction_ | **Women** | | | *p*_interaction_ |
| --- | --- | --- | --- | --- | --- | --- | --- | --- | --- | --- |
|  |  |  | OR (95% CI) | | |  | OR (95% CI) | | |  |
|  |  |  | T1 | T2 | T3 |  | T1 | T2 | T3 |  |
| **EPA** | ***FADS1***  **rs174546** | CC | 1.02  (0.56, 1.85) | 0.77  (0.42, 1.41) | 0.62  (0.34, 1.14) | 0.764 | 1.26  (0.65, 2.45) | 1.03  (0.50, 2.12) | 1.31  (0.65, 2.65) | 0.614 |
|  |  | TC/TT | 1.00  (Ref) | 0.67  (0.38, 1.19) | 0.82  (0.47, 1.44) |  | 1.00  (Ref) | 1.44  (0.74, 2.80) | 0.61  (0.29, 1.28) |  |
|  | ***FADS2***  **rs174583** | CC | 1.10  (0.60, 1.99) | 0.81  (0.44, 1.48) | 0.65  (0.35, 1.19) | 0.693 | 1.20  (0.62, 2.34) | 1.01  (0.49, 2.08) | 1.40  (0.70, 2.80) | 0.373 |
|  |  | TC/TT | 1.00  (Ref) | 0.69  (0.39, 1.21) | 0.83  (0.48, 1.46) |  | 1.00  (Ref) | 1.38  (0.72, 2.66) | 0.54  (0.26, 1.14) |  |
| **DHA** | ***FADS1***  **rs174546** | CC | 0.93  (0.51, 1.68) | 0.61  (0.34, 1.12) | 0.57  (0.31, 1.05) | 0.687 | 1.12  (0.58, 2.16) | 1.03  (0.51, 2.09) | 1.05  (0.52, 2.11) | 0.604 |
|  |  | TC/TT | 1.00  (Ref) | 0.55  (0.31, 0.97) | 0.67  (0.38, 1.18) |  | 1.00  (Ref) | 1.04  (0.53, 2.06) | 0.62  (0.31, 1.27) |  |
|  | ***FADS2***  **rs174583** | CC | 1.00  (0.55, 1.82) | 0.64  (0.35, 1.17) | 0.60  (0.32, 1.10) | 0.762 | 1.08  (0.56, 2.08) | 1.02  (0.50, 2.08) | 1.12  (0.56, 2.24) | 0.369 |
|  |  | TC/TT | 1.00  (Ref) | 0.57  (0.32, 0.99) | 0.68  (0.39, 1.20) |  | 1.00  (Ref) | 1.01  (0.52, 1.98) | 0.56  (0.27, 1.14) |  |
| **AA** | ***FADS1***  **rs174546** | CC | 0.91  (0.47, 1.75) | 0.60  (0.31, 1.14) | 1.46  (0.79, 2.71) | 0.071 | 0.79  (0.39, 1.61) | 1.36  (0.68, 2.71) | 1.77  (0.91, 3.46) | 0.162 |
|  |  | TC/TT | 1.00  (Ref) | 0.97  (0.53, 1.80) | 0.98  (0.55, 1.75) |  | 1.00  (Ref) | 1.32  (0.66, 2.63) | 0.83  (0.40, 1.71) |  |
|  | ***FADS2***  **rs174583** | CC | 1.05  (0.54, 2.03) | 0.63  (0.33, 1.21) | 1.58  (0.85, 2.92) | 0.127 | 0.70  (0.34, 1.44) | 1.44  (0.73, 2.85) | 1.73  (0.89, 3.38) | 0.095 |
|  |  | TC/TT | 1.00  (Ref) | 1.04  (0.57, 1.91) | 1.05  (0.59, 1.86) |  | 1.00  (Ref) | 1.14  (0.57, 2.28) | 0.78  (0.38, 1.60) |  |

ORs (odds ratios) and 95% CIs (95% confidence intervals) were adjusted for age, total caloric intake, body mass index, smoking (pack-years), drinking (ethanol amount), physical activity, *H. pylori* infection and family history of gastric cancer. *p*_interaction_ is the *p-*value for the interaction. EPA, eicosapentaenoic acid; DHA, docosahexaenoic acid; AA, arachidonic fatty acid.

**Supplementary Table 4**. Comparisons of general characteristics (n=1,710)

|  | Those  who were included  (n=1,464) | Those  who were excluded  (n=246) | p-value |
| --- | --- | --- | --- |
| Age, years | 52.92±9.40 | 53.72±8.80 | 0.211 |
| Women | 695 (47.47) | 126 (51.22) | 0.276 |
| Body mass index, kg/m^2^ | 23.78±2.97 | 23.48±2.82 | 0.176 |
| Smoking, pack-years^*^ | 11.80±66.85 | 7.54±13.80 | 0.012 |
| Drinking, ethanol amount, g/day^*^ | 12.11±26.11 | 9.23±21.94 | 0.002 |
| Helicobacter pylori, positive | 1,013 (69.19) | 162 (66.12) | 0.337 |
| Regular exercise | 713 (48.70) | 85 (42.93) | 0.127 |
| Family history of gastric cancer | 230 (15.71) | 30 (15.08) | 0.817 |
| **Dietary intake** |  |  |  |
| Total energy intake, kcal/day | 1772.49±605.48 | 1761.34±646.51 | 0.814 |
| Total fat intake, g/day | 32.81±19.53 | 33.18±19.62 | 0.807 |
| **n-3 polyunsaturated fatty acids** |  |  |  |
| α-linolenic acid (ALA), mg/day | 326.88±193.94 | 349.33±259.64 | 0.255 |
| Eicosapentaenoic acid (EPA), mg/day | 83.85±100.13 | 86.91±133.67 | 0.762 |
| Docosahexaenoic acid (DHA), mg/day | 169.46±220.04 | 178.73±297.69 | 0.681 |
| Sum of both EPA+DHA, mg/day | 253.30±319.730 | 265.64±431.16 | 0.706 |
| **n-6 polyunsaturated fatty acids** |  |  |  |
| Linoleic acid (LA), mg/day | 4207.81±2225.42 | 4301.63±2548.16 | 0.631 |
| Arachidonic acid (AA), mg/day | 14.02±12.70 | 13.91±16.01 | 0.929 |

Mean±S.D. or n (%); * p-value from the Wilcoxon test
